# Supplementary material for: Probing defect dynamics in monolayer MoS2 via noise nanospectroscopy
Source: Nat Commun. 2017 Dec 14;8:2121. doi: 10.1038/s41467-017-02297-3 (PMC5730608; doi:10.1038/s41467-017-02297-3)
Supplement: Supplementary file 1 — Supplementary Information [file 41467_2017_2297_MOESM1_ESM.pdf]

Supplementary Methods

Sample characterization

We use Raman spectroscopy and ac-mode atomic force microscopy (AFM) to verify that MoS_2 sheet samples prepared in this manner consist of monolayers, and to confirm sample quality and uniformity. Supplementary Fig. 1b presents Raman spectra collected at three different positions on the sample discussed in this article. The Stokes-shifts of the primary Raman-active phonon modes E_{2g} (386 cm^{-1}) and A_{1g} (404.5 cm^{-1}) are consistent with those reported for monolayer MoS_2 in the literature[1]; Raman spectra are identical across the flake. Supplementary Fig. 1c,d show AFM topography and phase lag images of the same MoS_2 flake. In ac-mode AFM, the phase lag between the sinusoidal excitation and the cantilever response provides a measure for the energy dissipated by the cantilever, i.e., the friction between the silicon tip and the sample surface[2]. Accordingly, a phase lag image provides information about the chemical homogeneity of the scanned surface. Taken together, our topography and phase lag images confirm that the MoS_2 surface is homogeneous and free from polymer residues. The measured height difference between MoS_2 and the gold substrate is $\sim$ 1.2 nm (Supplementary Fig. 1e), greater than the known interlayer spacing $c = 0.61$ nm of bulk MoS_2, i.e., we observe an apparent base height offset ~ 0.6 nm. Offsets of this magnitude are commonly seen in AFM measurements on surfaces consisting of dissimilar materials, such as MoS_2 deposited on SiO_2 (refs. 3–5). Raman spectra are collected on a WITec alpha-300 microscope, using a 100x objective and a Nd:YAG-laser of wavelength 532 nm, at a power of 0.5 mW; ac-mode AFM data are acquired on a Park Systems XE-7 AFM, using silicon cantilevers of spring constant 40 N/m and tip radius $r < 10$ nm.

Conductive atomic force microscopy (C-AFM) setup

Figure 1 of the main manuscript depicts the LF noise measurement setup. Samples are mounted inside the vacuum chamber of a Hitachi E-Sweep AFM, where they are clamped onto a temperature control unit consisting of a Joule heater and a cold finger connected to a liquid nitrogen bath. Samples are annealed at 80°C in vacuum for 2 hours prior to measurements; all measurements are performed in high vacuum ($\sim 5 \times 10^{-6}$ torr). We perform C-AFM measurements using an ElectriMulti75 AFM cantilever with a Pt-coated tip of radius $r < 25$ nm, using a contact force $\sim$ 20 nN. We use the internal voltage source of Hitachi E-Sweep to supply a dc-bias voltage between the AFM tip and the gold substrate. The resulting

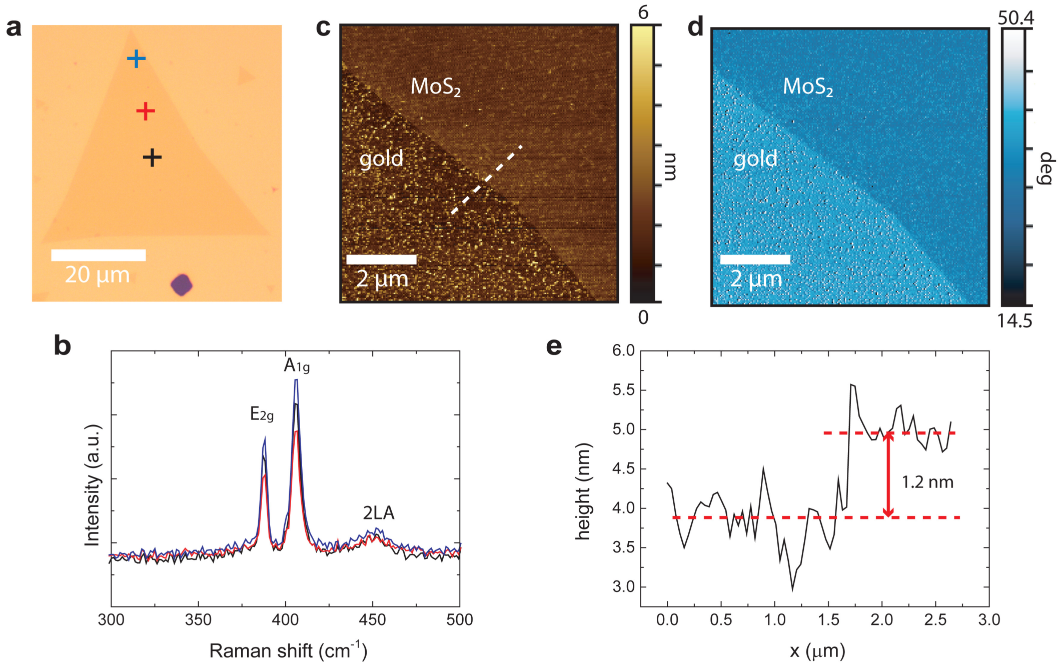

Supplementary Figure 1: **Sample characterization.** (**a**) Brightfield photograph of CVD-grown monolayer MoS_2 sample deposited on gold substrate. (**b**) Raman spectra acquired at positions indicated in panel **a**. The spectra confirm the sample's high crystallinity and uniformity. (**c**) Tapping mode AFM topography and (**d**) phase lag image. The sample surface is smooth and free of polymer contamination. (**e**) Height trace at the position indicated in panel **c**.

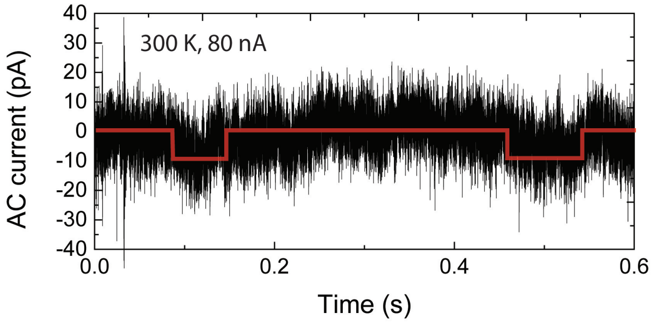

Supplementary Figure 2: **Typical measured ac-current trace.** Fast current fluctuations, due to switching of vacancy defects between $V_S(0) \leftrightarrow V_S(-1)$ charge states, are superimposed onto rare switching events between high and low current states (red line), due to $V_S(-1) \leftrightarrow V_S(-2)$ occurring at the much lower characteristic frequency $f_\alpha \simeq 4$ Hz.

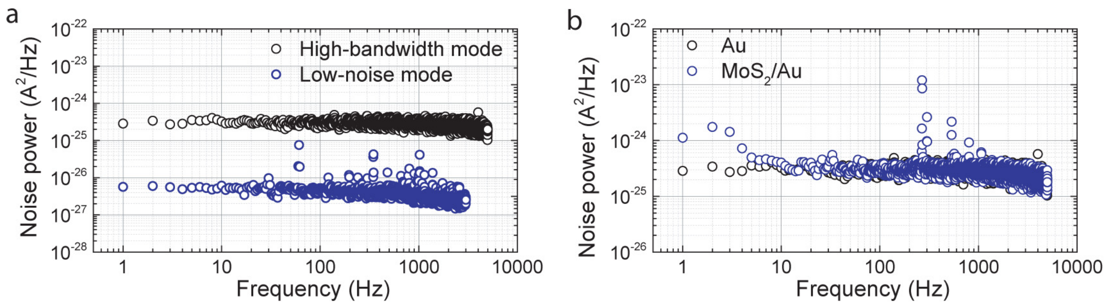

Supplementary Figure 3: **Determination of noise floor.** (**a**) Current noise power spectra measured on a bare gold substrate at $T = 300$ K, $I_{\mathrm{bias}} = 5$ nA, using the preamplifier's 'low noise' mode (blue) and 'high bandwidth' mode (black). The noise floor determined in this manner coincides closely with manufacturer specifications. (**b**) Extrinsic noise introduced by temperature control setup. LF noise power spectra of monolayer MoS_2 deposited on gold (blue) and reference spectrum measured on the gold substrate alone (black). Both spectra were acquired at $T = 300$ K, $I_{\mathrm{bias}} = 1$ nA, using the preamplifier's 'high bandwidth' mode. Importantly, the narrow peaks induced by the temperature control setup do not distort the overall spectral shape. The rise visible at < 10 Hz for monolayer MoS_2 reflects the Lorentzian peak at $f_\alpha \simeq 4$ Hz.

current is sensed through a noise measurement system consisting of a series resistor (100 MΩ), current preamplifier (SR570, Stanford Research Systems), and a data acquisition system (National Instruments DAQ-4431) that records both the dc-current as well as its ac-fluctuations, at an acquisition rate 100 kHz (ref. 6). The series resistor prevents the AFM tip from overheating by keeping the current below 100 nA. This work is based on a total of 285 spectra, acquired at different positions on the same MoS_2 sample, using a single AFM cantilever. Spectra acquired on other MoS_2 samples (not shown in this work) were qualitatively identical. For the acquisition of LF noise spectra over the temperature range $150-300$ K, we utilize a temperature control unit that forms a built-in part of the AFM instrument used in this study. Use of this unit comes at the cost of increased noise introduced into acquired spectra at specific frequencies.

Discussion of noise floor

The lowest noise signal detectable by our setup is determined either by the system noise of the circuitry employed, or by the thermal Johnson-Nyquist noise in the sample, depending on which is greater.

According to its manufacturer specifications, the current preamplifier of our setup (SR570, Stanford Research Systems) has a noise floor of $4 \cdot 10^{-27}$ A^2/Hz in its 'low noise' mode, and $2.5 \cdot 10^{-25}$ A^2/Hz in its 'high bandwidth' mode. These values agree well with the noise profile we measure using a bare gold substrate for both preamplifier modes, shown in Supplementary Fig. 3a. We thus establish that system noise of our setup is limited by preamplifier noise, with negligible contributions from other components;

this measured noise floor enters our data analysis as constant offset C in Eqn. (1) [main text].

The current noise generated in an Ohmic resistor with resistance R due to thermal Johnson-Nyquist noise is

$$S_I = \frac{4k_{\mathrm{B}}T}{R}, \tag{1}$$

where k_{B} denotes the Boltzmann constant and T is absolute temperature. In our setup, resistance is dominated by a series resistor ($R = 100$ MΩ). For the highest temperature used in our measurements, $T = 300$ K, we calculate the contribution of Johnson-Nyquist as $S_I = 1.66 \cdot 10^{-28}$ A^2/Hz. This value is far below the noise floor imposed by the current preamplifier, regardless of whether the 'low noise' or the 'high bandwidth' mode is used, and thus we do not consider Johnson-Nyquist noise in the evaluation of our data. We remark that due to the use of a 100 MΩ series resistor, the overall resistance of our setup will change only minimally ($< 10\,\%$) when incorporating the MoS_2 monolayer sample or when varying sample temperature, such that our neglect of Johnson-Nyquist noise remains justified in those circumstances. Supplementary Fig. 3b illustrates this point by comparing a noise spectra acquired on an MoS_2 monolayer deposited on a gold substrate with the spectrum of the gold substrate alone.

Importantly, having thus established that thermal Johnson-Nyquist noise is below our noise floor in all measurement configurations used in our experiments, we show clearly that any noise power contributions in excess of the noise floor originate from the MoS_2 monolayer sample.

Discussion of measurement bandwidth

Here, we discuss the possibility of spectral distortion of measured noise power spectra due to limited bandwidth of the circuit. Coaxial cables feature a stray capacitance of typically 100 pF/m. In our measurements, we employ a BNC cable of length 20 cm, such that $C \simeq 20$ pF. Together with the large value of our series resistor ($R = 100$ MΩ), this would lead to a cut-off frequency of the order 1 kHz in an uncorrected circuit. This issue is resolved by using a preamplifier that employs internal circuitry designed to preserve the signal amplitude and phase in the presence of a large source resistance and stray capacitance (SR570, Stanford Research Systems). According to manufacturer specifications, the preamplifier's rated bandwidth (in which the response is flat) is 20 kHz for the settings chosen in our variable-temperature experiments ('high bandwidth' mode, with sensitivity 100 nA/V), for a stray capacitance $C \leq 100$ pF and provided that the source resistance $R \gg 10$ MΩ. Since these conditions are fulfilled in our setup, and

given the limited frequency range of our study ($f \leq 5$ kHz), no spectral distortions of our noise spectra due to circuit bandwidth are expected. For the preamplifier's 'low noise' mode used for measurements at room temperature only [Fig. 3b,d in main text], manufacturer specifications guarantee a rated bandwidth of 2 kHz. We truncate corresponding spectra at the experimentally determined 3 dB-point at 3 kHz.

Influence of extrinsic noise

The measurement's sensitivity is dictated by the noise characteristics of the current preamplifier. For room temperature measurements, where no heater is required, the amplifier's 'low noise' mode determines the noise floor of $4 \cdot 10^{-27}$ A^2/Hz; at lower temperatures, where a heater is required for temperature regulation, noise introduced by the temperature control system requires using the amplifier's 'high bandwidth' mode, leading to a noise floor of $2.5 \cdot 10^{-25}$ A^2/Hz. This noise leads to the introduction of sharp peaks into the power spectrum, at a frequency $\simeq$ 300 Hz and its overtones, as well as a smaller peak at $\simeq$ 30 Hz. While these peaks visually dominate the high-frequency portion of the noise power spectrum, the shape of the original noise spectra is preserved, as shown in Supplementary Fig. 3b, where a noise spectrum on MoS_2 at low bias current is compared with the noise floor due to the preamplifier; no distortion of the signal envelope or increase in the noise floor are observed.

Data processing

Each noise spectrum is obtained in the following manner: we acquire 50 subsequent time-domain current traces of 1 second duration, perform a Fourier transform on each of these traces, and then average the 50 noise spectra computed in this fashion for an improved signal-to-noise ratio. To extract relevant physical parameters, we perform unweighted least-squares fits to Eqn. (1) provided in the main text; for data sets acquired using temperature control that are affected by the associated excess noise, we exclude narrow frequency intervals around the sharp peaks due to extrinsic noise by evaluating derivative values. Numerical values of fit results depend only weakly on whether the effect of extrinsic noise is excluded in this manner or not.

The main effect of extrinsic noise due to the temperature control system is a greater spread in extracted fit parameter values, leading to an increased error estimate. This is illustrated in Supplementary Fig. 4, showing the values of characteristic frequencies (f_α, f_β) extracted from noise power spectra at

temperature range of 150 to 300 K as a function of bias current. The numerical values of f_α, f_β show no apparent trend as the bias current is varied.

Supplementary Note 1. Predicted dc-bias current dependence of noise power spectra for different sources of current fluctuations

In the following, we discuss how different sources of current fluctuations affect the prefactors $A_\alpha(\bar{I})$ and $A_\beta(\bar{I})$ of Eqn. (1) in the main text. We use the notation I for the instantaneous current flowing in the sample, $\bar{I}$ to designate the mean current (which in our case is identical to the dc-bias current I_{bias}), and ΔI to denote the current fluctuation amplitude. Since $|\Delta I| \ll |I|$, we equate $\bar{I} \simeq I$.

For a single fluctuation frequency, Eqn. (1) [main text] becomes

$$S_I = \frac{A(\bar{I})\bar{I}^2}{1+\left(\frac{f}{f_0}\right)^2}. \tag{2}$$

By definition, the noise power $S_I \propto (\Delta I)^2$, where ΔI denotes the current fluctuation amplitude. Equating this to Supplementary Eqn. (2) yields

$$A(\bar{I}) \propto \left(\frac{\Delta I}{\bar{I}}\right)^2. \tag{3}$$

In the scenario of an FET geometry, where I describes a drift current, the following cases are normally considered: if current fluctuations are dominated by mobility fluctuations, $A(\bar{I})$ is independent of $\bar{I}$. In contrast, if current fluctuations are dominated by carrier number fluctuations (due to trapping/detrapping of electrons), $A(\bar{I}) \propto 1/\bar{I}^2$ (ref. 7).

The sample geometry studied in our work represents the separate scenario of a tunnel current,

$$I \propto V\rho\,\mathrm{e}^{-\kappa d}, \text{ with } \kappa = \frac{\sqrt{2mE}}{\hbar}. \tag{4}$$

Here, V denotes the applied bias voltage at the AFM tip, and ρ is the density of states (DOS) at the Fermi level (i.e., the DOS of the metallic substrate). The quantities d and E are the width and height of the tunneling potential barrier, which in our sample is provided by the MoS_2 layer.

Differentiation of Supplementary Eqn. (4) and division by I yield

$$\frac{\Delta I}{I} = \frac{\Delta V}{V} - \frac{\Delta E}{\hbar}\sqrt{\frac{m}{2E}}. \tag{5}$$

We first consider the case that current fluctuations are dominated by fluctuations ΔE of the barrier height E. In the presence of a vacancy V_S, E represents the energy difference between the Fermi level

and the defect states (mid-gap states e shown in Fig. 2b in the main manuscript). Trapping of an electron on V_S will occupy the lowest available defect state and lead to an increase ΔE of the effective tunneling barrier height, due to Coulomb repulsion-induced upshifting of the remaining defect states. It follows that

$$A(\bar{I}) \propto \left(\frac{\Delta I}{\bar{I}}\right)^2 = \frac{m}{2\hbar^2}\frac{(\Delta E)^2}{E}. \tag{6}$$

None of the terms on the right hand side depend on $\bar{I}$, thus $A(\bar{I})$ is independent of $\bar{I}$ in this case.

Next we address the case that current fluctuations are dominated by fluctuations ΔV of the effective bias voltage V. Trapping/detrapping of electrons at vacancies will lead to a voltage variation $\Delta V \propto N_{\text{trap}}$, the latter designating the number of vacancies in the probed area. Thus,

$$A(\bar{I}) \propto \left(\frac{\Delta I}{\bar{I}}\right)^2 = \left(\frac{\Delta V}{V}\right)^2 \propto \frac{N_{\text{trap}}^2}{V^2} \propto \frac{1}{\bar{I}^2}, \tag{7}$$

where the last step, $V \propto I$, follows from Supplementary Eqn. (4). We remark that this dependence $A(\bar{I}) \propto 1/\bar{I}^2$ is identical to the one found for carrier number fluctuation-dominated current noise in an FET geometry, as mentioned above. The two scenarios are in fact analogous; this can be seen by grouping factors in Supplementary Eqn. (4), $n = V\rho$, such that n quantifies the number of accessible states. It follows that $\Delta V/V = \Delta n/n$, i.e., current noise is dominated by fluctuations in the number of accessible electron states.

Supplementary Note 2. Experimentally observed dc-bias current dependence of noise power spectra

We acquire noise power spectra over the dc-bias current range $0.1-100$ nA, for temperatures $150-300$ K. All acquired noise power spectra are described well by Eqn. (1) provided in the main text, comprising Lorentzian peaks centered at characteristic frequencies f_α and f_β.

In Supplementary Fig. 4, we show the frequencies f_α and f_β obtained by fits of Eqn. (1) [main text] to spectral data, for the entire range of bias current and temperatures covered in our experiments. Within the scatter of data points, neither f_α nor f_β vary systematically as a function of I_{bias}. This observation indicates that the low-injection limit of the Shockley-Read-Hall model is applicable, justifying the use of Eqn. (2) in the main manuscript.

In contrast, we observe a qualitatively different behaviour for the normalized noise amplitudes $A_\alpha(I_{\text{bias}})$ and $A_\beta(I_{\text{bias}})$ that result from the same fit procedure [Supplementary Fig. 5]. As I_{bias} in-

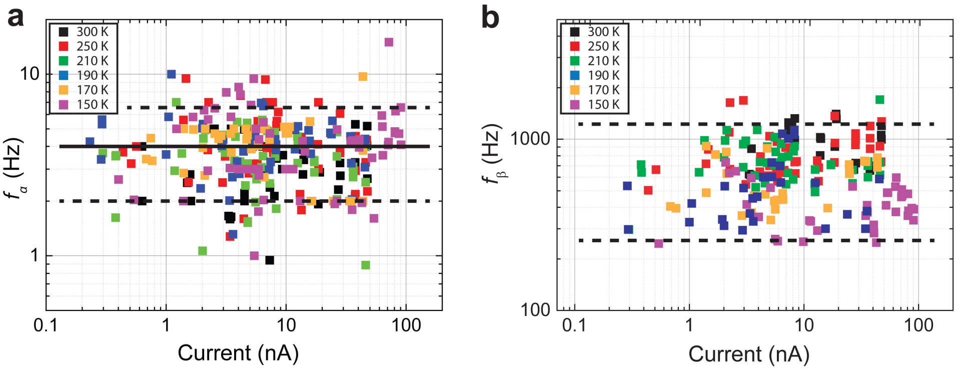

Supplementary Figure 4: **Evolution of characteristic frequencies f_α and f_β with bias current, for the temperature range 150 - 300 K.** (**a**) f_α, (**b**) f_β. Numerical values of f_α, f_β are obtained by fits of Eqn. (1) [main text] to spectral data. We observe that $f_\alpha \simeq 4$ Hz is constant within the scatter of individual data points, showing no dependence on temperature or bias current. Guides drawn at 2 and 6.5 Hz indicate the standard deviation of the set of data points. In contrast, f_β decreases with decreasing temperature [see Fig. 3f and discussion in main text]; however, also in this case no systematic trend with I_{bias} is apparent. As guides-to-the-eye, we indicate the lower standard deviation of $f_\beta(150\ \mathrm{K})$ at 250 Hz, and the upper standard deviation of $f_\beta(300\ \mathrm{K})$ at 1200 Hz. [See Fig. 3f in main manuscript for the standard deviations of all data points.]

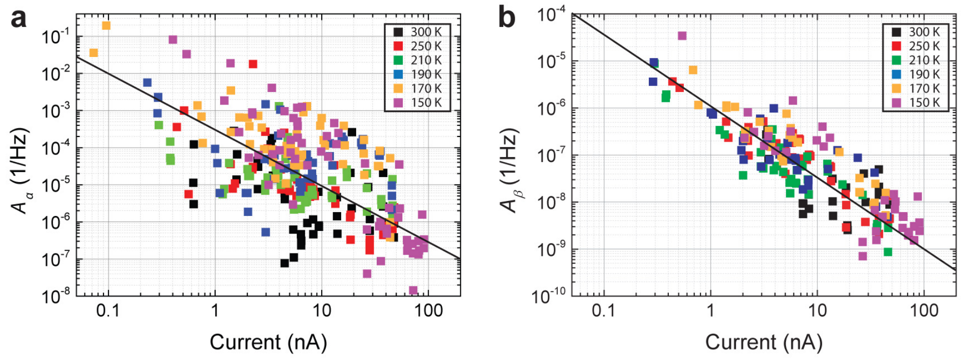

Supplementary Figure 5: **Evolution of normalized noise power amplitudes $A_\alpha(I_{\mathrm{bias}})$ and $A_\beta(I_{\mathrm{bias}})$ with bias current, for the temperature range 150 - 300 K.** (**a**) $A_\alpha(I_{\mathrm{bias}})$, (**b**) $A_\beta(I_{\mathrm{bias}})$. The noise power amplitudes are defined by Eqn. (1) [main text], and numerical values are evaluated by fitting that equation to spectral data. Neither A_α nor A_β exhibit a systematic variation with temperature. In contrast, both quantities decrease over ~ 4 orders of magnitude with increasing bias current. For the higher-frequency component, a clear relationship $A_\beta(I_{\mathrm{bias}}) \propto (I_{\mathrm{bias}})^{-1.5\pm0.25}$ is observed [black line in panel **b**]. For the lower-frequency component A_α, relative scatter of data points precludes extraction of a precise scaling relationship; we indicate $A_\alpha(I_{\mathrm{bias}}) \propto (I_{\mathrm{bias}})^{-1.5}$ for reference [black line in panel **b**].

creases, both normalized noise amplitudes diminish by approximately 4 orders of magnitude. For the higher-frequency noise contribution, we extract the scaling behaviour $A_\beta(I_\mathrm{bias}) \propto (I_\mathrm{bias})^{-1.5\pm0.25}$ [Supplementary Fig. 5b]. The case of $A_\alpha(I_\mathrm{bias})$ [Supplementary Fig. 5a] is less clear due to significant scatter of the data points; nonetheless, the trend observed in this data set is also consistent with a scaling $(I_\mathrm{bias})^{-1.5}$.

As discussed in Supplementary Note 1, for current noise dominated by variations of the tunneling barrier height, we expect that $A(I_\mathrm{bias}) = \mathrm{const}$ [Supplementary Eqn. (6)], whereas in the case that fluctuations in the number of accessible electron states are the dominant noise mechanism, it is predicted that $A(I_\mathrm{bias}) \propto (I_\mathrm{bias})^{-2}$ [Supplementary Eqn. (7)]. Our empirical finding that both $A_\alpha(I_\mathrm{bias})$ and $A_\beta(I_\mathrm{bias})$ scale approximately as $A(I_\mathrm{bias}) \propto (I_\mathrm{bias})^{-1.5}$ provides evidence that both mechanisms contribute strongly to the observed current noise in monolayer MoS_2.

Supplementary Note 3. Discussion of spectral shape: Lorentzian vs. $1/f$

Spectra acquired on our samples at room temperature, using the current preamplifier's 'low noise' mode [Fig. 3b,d in main manuscript] unambiguously exhibit two separate Lorentzian contributions at characteristic frequencies f_α and f_β, by virtue of signal amplitudes that far exceed the noise floor. In particular, by highlighting the discrepancy between the slope and overall shape of our spectra on the one hand, and the $1/f$ dependence expected in cases where flicker noise is dominant on the other hand [black line in Fig. 3d], we clearly exclude the alternative possibility that our spectra could be explained by $1/f$ noise, or by a superposition of $1/f$ noise and a Lorentzian component.

By continuity, we expect that LF noise spectra acquired in our variable-temperature measurements comprise two separate Lorentzian components as well. Nonetheless, we wish to present independent evidence for this view. In our variable-temperature experiments, extrinsic noise peaks require that we use the current preamplifier's 'high bandwidth' mode (see Supplementary Methods), which results in an enhancement of the noise floor by a factor ~ 60. Supplementary Fig. 6 shows representative noise power spectra collected in this configuration at 300, 210, and 150 K.

For low dc-bias current values [Supplementary Fig. 6a,c,e], the high-frequency f_β-component of acquired spectra is either below or comparable to the noise floor amplitude, precluding clear statements about the spectral shape in these cases. However, at higher bias currents [Supplementary Fig. 6b,d,f],

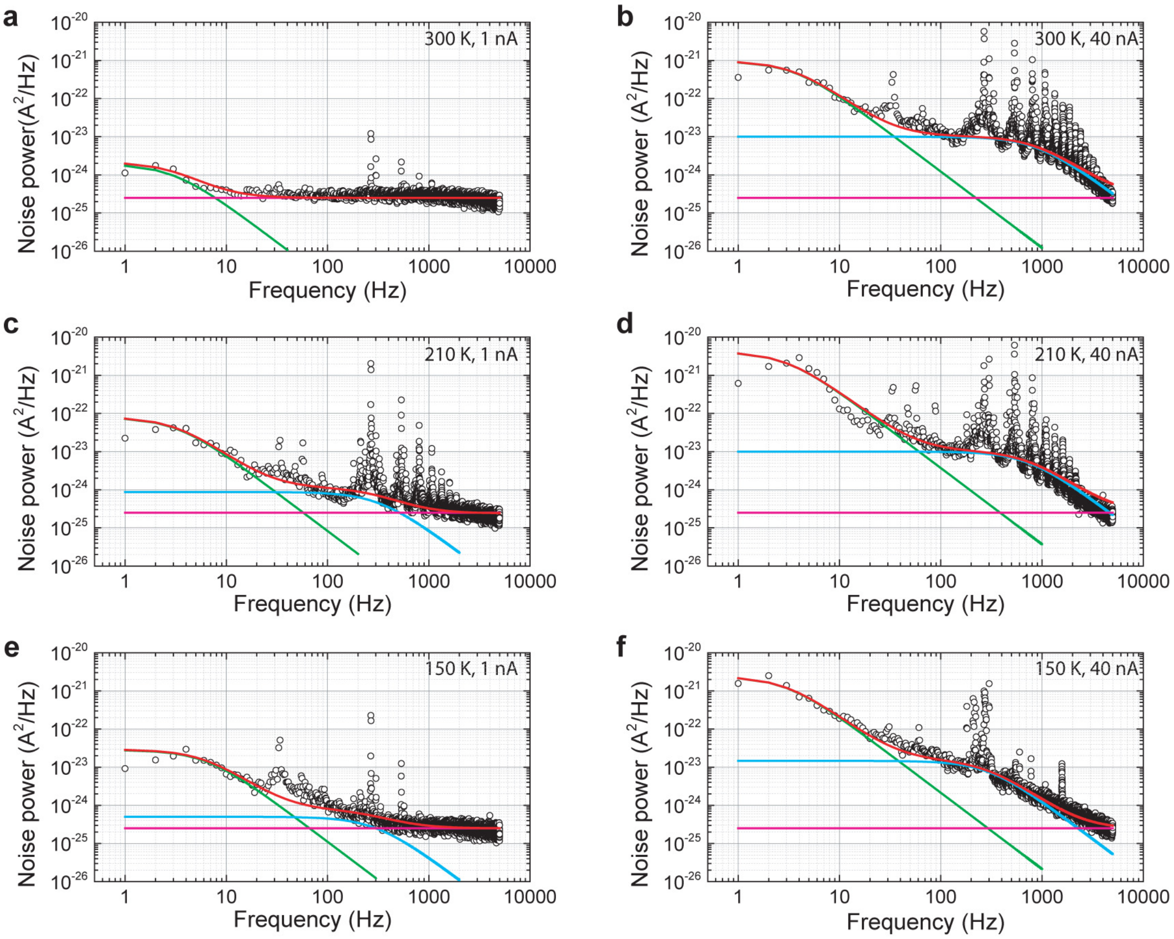

Supplementary Figure 6: **LF noise spectra acquired in variable-temperature measurements.** Use of the AFM's built-in temperature control setup induces several sharp noise peaks into the spectra. (**a**) $T = 300$ K, $I_{\text{bias}} = 1$ nA, and (**b**) $I_{\text{bias}} = 40$ nA. [Spectra acquired at 300 K without temperature control are shown in Figure 3b,d in main manuscript.] (**c**) $T = 210$ K, $I_{\text{bias}} = 1$ nA, and (**d**) $I_{\text{bias}} = 40$ nA. (**e**) $T = 150$ K, $I_{\text{bias}} = 1$ nA, and (**f**) $I_{\text{bias}} = 40$ nA. The presence of extrinsic noise peaks requires using the pre-amplifier's 'high bandwidth' mode, leading to a higher noise floor.

both the low-frequency f_α- and the high-frequency f_β-components are present. In spectra collected at 300 and 210 K [Supplementary Fig. 6b,d], notwithstanding the presence of sharp extrinsic noise peaks, the spectral shape visually appears quite distinct from that of a $1/f$ spectrum. For a more quantitative statement, we compare the alternatives (1) two Lorentzian functions plus constant background, vs (2) a single $1/f$ function plus constant background, by computing least squares fits of these candidate functions to the spectrum shown in Supplementary Fig. 6b. This procedure leads to a ratio of residuals $R^2(2 \times \text{Lorenzian})/R^2(1/f) \simeq 0.02$, clearly favouring an explanation in terms of Lorentzian components.

At the lowest temperature $T = 150$ K studied in our experiments, a clear visual distinction between our measured spectra [Supplementary Fig. 6f] and a $1/f$ spectral shape is no longer possible. Nonetheless, the ratio of residuals obtained in this case, $R^2(2 \times \text{Lorenzian})/R^2(1/f) \simeq 0.3$, does not support an explanation in terms of a $1/f$ spectrum either.

Supplementary Note 4. Discussion of monovacancy V_S energy level diagram

Figure 2d [main manuscript] shows a schematic energy level diagram of the monovacancy V_S, summarizing the conclusions drawn from our experimental data. Here, we gather the evidence underpinning that energy level diagram in the form of a table for convenient review:

- In noise spectra acquired anywhere on our MoS_2 monolayer samples, we observe two switching processes (at characteristic frequencies $f_\alpha \simeq 4$ Hz, $f_\beta \simeq 1$ kHz). From this observation, we infer the presence of 3 different charge configurations of V_S.

- From the temperature dependence of f_α, f_β, we calculate the associated energy barriers E_α, E_β (and capture cross sections $\sigma_\alpha^{(0)}, \sigma_\beta^{(0)}$) via Eqn. (2) [main text]. The energy barriers $E_\alpha < 15$ meV, $E_\beta \simeq 23$ meV are schematically indicated in Fig. 2d.

- The inferred capture cross sections differ by about 3 orders of magnitude, $\sigma_\alpha^{(0)} \ll \sigma_\beta^{(0)}$. Since noise spectra are acquired using an electron current, a neutral defect will have a much larger cross section than a negatively charged defect. Moreover, $\sigma_\alpha^{(0)} \approx 10^{-24}$–$10^{-23}$ cm^2 and $\sigma_\beta^{(0)} \approx 10^{-21}$–$10^{-20}$ cm^2 have orders of magnitude typical for negatively charged and neutral defects in SiO_2, respectively[8]. Furthermore, we can exclude the presence of positive charge states, since these are

known to have capture cross sections far larger than those observed in our measurements[8,9].

Up to this point, we have inferred that fast switching ($f_\beta \simeq 1$ kHz) occurs between a neutral state and a negatively charged state, whereas slow switching ($f_\alpha \simeq 4$ Hz) occurs between two negatively charged states. We identify these states with V_S(0), V_S(-1), and V_S(-2).

- In order to deduce the relative energy levels of the V_S charge states, we inspect raw noise current traces to infer the duty cycles associated with different charge states, i.e., what fraction of time is spent in the charge state. For switching on short timescales [$\sim$ 1 msec, associated with f_β, see Fig. 3a,b], we do not observe a preferred level, from which we conclude that V_S(0) and V_S(-1) have approximately equal energies. In contrast, switching on long time scales [$\sim$ 100 msec, associated with f_α, see Supplementary Fig. 2], we observe that current values dwell in the higher current state [i.e., V_S(-1)] for a majority of the time; from this we conclude that V_S(-2) is a higher energy state than V_S(-1).

This concludes the observations upon which we base the schematic energy diagram shown in Fig. 2d. We remark that based on our measurements we cannot discriminate whether V_S(0) or V_S(-1) is the ground state for the Fermi energy E_F realized in our experiments.

The energy level diagram constructed by us in this way is consistent with theoretical predictions that for E_F close to the conduction band minimum, V_S(0) and V_S(-1) have similar formation energies, whereas the formation energy of V_S(-2) is significantly higher[10,11].

Supplementary Note 5. Discussion of MoS_2 defect type dominating LF noise

In this work, we attribute the tunneling current switching events observed in our measured LF noise spectra to the presence of sulfur monovacancies (V_S), which is the most common type of lattice defects in MoS_2; divacany defects (V_{S2}, V_{2S}), while expected to be present at significantly lower areal densities, may also contribute and are a possible origin of the weak variability observed in our measured spectra. In contrast, we do not assign a role to other defect types known to exist in MoS_2, on the basis that these defects are expected to be far rarer than V_S defects in our CVD-MoS_2 samples. In this section, we discuss the evidence that supports this notion.

We begin by considering the formation energies of different types of native point-like defects in monolayer MoS_2. Supplementary Table 1 lists theoretically predicted formation energies of a variety of defect types that have been reported in the literature[10–14], calculated for the assumption of molybdenum-rich conditions that most closely resemble experimental conditions during the CVD-growth of MoS_2. The table omits literature reports that calculate defect formation energies for other conditions[15,16], however these authors come to qualitatively similar results as well.

It is immediately apparent from Supplementary Table 1 that the defects with the lowest formation energies, i.e., the defects that have the highest formation probability, are the sulfur monovacancy V_S and the sulfur adatom defect S_{ad} [also referred to as sulfur interstitial (S_i) in the literature[10,11]]. We can immediately exclude S_{ad} from further consideration, because this defect does not lead to the creation of energy states within the bandgap of MoS_2 (refs. 10,11,15) and thus it cannot contribute to charge switching events detected by LF noise measurements.

Supplementary Table 1 further shows that the formation of sulfur divacancy defects (V_{S2}) is not preferred over the formation of monovacancies, because $E(V_{S2}) \simeq 2E(V_S)$ (ref. 12, also found by refs. 15,16), and thus this type of defect should be rare. This accords with the experimental finding that both types of divacany defects (V_{S2}, V_{2S}) are far less common that monovacancies[12,16–18], and defects involving two neighbouring divacancies (V_{2S2}) are yet less common[16] (see Supplementary Table 2).

All further defects listed in Supplementary Table 1, comprising molybdenum interstitials (Mo_i), molybdenum vacancies (V_{Mo}), as well as a variety of anti-site and larger vacancy defects, possess far larger formation energies that V_S, and thus they are expected to be present in MoS_2 samples only at very low densities. This expectation has been largely confirmed experimentally; Supplementary Table 2 lists the relative observation rates of different defect types in MoS_2 reported in literature reports of STM and TEM studies[12,16,18]. From the table, it is clear that anti-site and large vacancy defects are observed very rarely compared to V_S. The only exception to this rule is a single report of moderately high densities of molybdenum vacancies V_{Mo} in monolayer MoS_2 (ref. 16). Given that it is at variance with other work that explicitly states that V_{Mo} defects are not observed[12], we suggest the possibility that this report is in error, especially since V_{Mo} is not expected to be stable, due to $E(V_{MoS3}) < E(V_{Mo})$ (refs. 11,12, see Supplementary Table 1), even more so under electron irradiation during TEM measurements.

We now discuss the areal density of sulfur monovacancies in MoS_2. Supplementary Table 3 summarizes experimentally determined V_S densities reported in the literature[12,16,18–22]. Given the wide spread of reported values, spanning three orders of magnitude, it is clear that there is no universal agreement on the issue. Several factors likely contribute to the disparity. Firstly, TEM studies typically overestimate the density of V_S due to vacancy creation by electron impact, especially at high kinetic energies; theoretical and experimental work has demonstrated that for an acceleration voltage of 80 kV, significant numbers of V_S are generated[17]. Secondly, previous work[20,23] has demonstrated that the composition of *natural* MoS_2 samples is extremely variable, both between samples and within samples, which is reflected in an equally uneven defect distribution within samples; these authors reported the observation of stoichiometry variations MoS_x, $x = 1.8 - 2.3$, within samples of geological origin[20,23], whereas other such samples had uniform stoichiometry[23]. Accordingly, it is difficult to assess to what degree densities of V_S reported in the literature for exfoliated natural MoS_2 are representative.

Next, we address the question what areal density of V_S defects to assume for the CVD-MoS_2 monolayer samples studied in this work by noise spectroscopy. Previous work from our laboratory[22] has found a V_S density of $(4 \pm 1) \cdot 10^{11}$ cm^{-2}, in CVD-MoS_2 synthesized with closely similar growth parameters, such that this value is likely to best represent the properties of our present samples.

A separate argument allows us to exclude defects other than sulfur vacancies V_S (and divacancies V_{S2}, V_{2S}) as significant contributors to our measured LF noise spectra. The central observation for this argument is that the noise spectra acquired in this work are homogeneous as a function of position on the MoS_2 sample, i.e., the spectral shape is invariant and characteristic frequencies f_α, f_β exhibit a relatively narrow spread (see Supplementary Figure 4). This observation indicates that any defect species that contributes to current switching processes is required to be present at an average density ρ_{defect} greater than a single defect per AFM tip/sample contact area. With the known radius of the metallized AFM tip, $r < 25$ nm, and assuming as worst-case scenario that the entire tip cross-section contributes to the detected current, we can place a lower bound on the average defect density, $\rho_{\mathrm{defect}} \gg 1/(\pi r^2) > 5 \cdot 10^{10}$ cm^{-2}. Comparing this threshold to the defect densities summarized in Supplementary Table 2, we can exclude all types of point-like defects with the exception of V_S (and sulfur divacancies) as possible candidates. (We have discussed above that we exclude V_{Mo} as a candidate, because we consider the

report of its experimental observation to be erroneous.)

We note explicitly that this argument does not permit us to exclude the possibility that two types of sulfur divacancies (V_{S2}, V_{2S}) coexist with monovacancies V_S in our samples. The divacancy V_{S2} (vacant S-sites in opposite S-sheets) has been shown to have mid-gap energy levels that are virtually indistinguishable from V_S (ref. 12,15). For the divacancy V_{2S} (vacant S-sites in the same sheet), additional levels appear inside the band gap at energies above the e-level[15,18]; it is possible that the presence of V_{2S} defects contributes to the weak variability of characteristic frequencies f_α, f_β that we observe.

Up to this point, we have established that amongst *native* point defects in MoS_2 that lead to the formation of states within the band gap, monovacancies V_S are expected to dominate the measured LF noise signals. Regarding the role of *impurity* defects, a wide variety of metallic element impurities has been experimentally detected both in natural MoS_2 and, to a lesser degree, in synthetic MoS_2 crystals[23]. In both cases, the most abundant impurity element was found to be bismuth. Converting the bismuth weight fraction reported in these studies leads to an areal density of Bi-impurities in monolayer MoS_2 of $\sim 2 \cdot 10^{10}$ cm^{-2} in the case of natural MoS_2, and $\sim 4 \cdot 10^{8}$ cm^{-2} for synthetic MoS_2. The impurity levels detected for other elements were significantly lower in both cases[23]. Given that areal densities fall below our threshold, $\rho_{\text{defect}} \gg 5 \cdot 10^{10}$ cm^{-2}, especially for MoS_2 of synthetic origin which we assume to be more representative for the CVD-grown MoS_2 samples utilized in our work, we can rule out that metallic element impurities contribute significantly to the noise spectra observed in our studies.

We conclude with the question whether reaction of MoS_2 with ambient molecular oxygen can potentially affect the measured LF noise spectra. Theoretical work has shown that both the dissociative adsorption of an oxygen atom on top of a S-site (O_{ad}) [ref. 24] as well as the dissociative filling of V_S by an oxygen atom (O_S) [ref. 17,24] are indeed thermodynamically favourable, albeit impeded by kinetic barriers[24]. However, neither O_{ad} nor O_S defects lead to the creation of states within the band gap[17,24], and thus these particular impurity defects cannot account for the tunneling current switching observed in our measurements.

In order to minimize the influence of adsorbed ambient species in our measurements, we anneal the MoS_2 sample in high vacuum, $P \sim 5 \times 10^{-6}$ torr, prior to C-AFM measurements (see Methods). In a control experiment, we repeated the C-AFM measurement on the same sample in ambient atmosphere;

LF noise power spectra resulting from the two measurements performed in high vacuum and in ambient were virtually identical.

Taken together, the above summary of literature reports and our own experimental observations provide strong evidence that our LF noise measurements do indeed reflect the dynamics of sulfur monovacancy defects.

	defect type	defect generates deep gap states?	Zhou *et al.* (ref. 12, Fig. 2c)	Noh *et al.* (ref. 10, Fig. 14)	Komsa *et al.* (ref. 11, Fig. 6a)	Liu *et al.* (refs. 13,14)
S monovacancy	V_{S}	yes [10–18]	1.5 eV	1.5 eV	1.3 eV	0.95 eV
S adatom on top of S-site	S_{ad}	none [10,11,15]	-	2.4 eV	2.4 eV	-
neighbouring S vacancies (in opposite S-sheets)	V_{S2}	yes [12,15]	3.0 eV	-	-	-
neighbouring S vacancies (in same S-sheet)	V_{2S}	yes [15,18]	-	-	-	-
split Mo interstitial	Mo_{i}	yes [10,11]	-	4.3 eV	3.1 eV	-
	Mo_{S}	yes [11,16]	-	-	3.4 eV	-
	Mo_{S2}	yes [12,16]<br>none [11]	5.7 eV	-	6.9 eV	-
	V_{MoS3}	yes [12]	6.5 eV	-	6.0 eV	-
	V_{Mo}	yes [10,11,15]	7.7 eV	7.8 eV	7.8 eV	8.02 eV
	V_{MoS2}	-	8.3 eV	-	-	-
	S_{Mo}	yes [11]	-	-	8.8 eV	-
	V_{MoS6}	yes [12]	11.3 eV	-	10.0 eV	-
	$S2_{Mo}$	yes [11,12]	11.5 eV	-	$>$ 10 eV	-

Supplementary Table 1: Theoretically predicted formation energies of various types of point-like lattice defects in monolayer MoS_2, reported in refs. 10–14. The table includes only reports that provide defect formation energies for monolayer MoS_2 under molybdenum-rich conditions, i.e., closest to experimental conditions during CVD-growth. Listed energies are for defects' neutral charge states. The presence of states introduced within the bandgap is a precondition to detect defects by noise spectroscopy. A brief description of the defect structure is added where abbreviated symbols are not self-explanatory.

	Vancsó *et al.* (ref. 18)	Hong *et al.* (ref. 16, Fig. 1d)		Zhou *et al.* (ref. 12)
technique	STM	TEM		TEM
sample	exfoliated synthetic MoS_2 monolayer	exfoliated natural MoS_2 monolayer	CVD-MoS_2 monolayer	CVD-MoS_2 monolayer
V_S	$5 \cdot 10^{12} - 5 \cdot 10^{13}$ cm^{-2}	$(1.1 \pm 0.4) \cdot 10^{13}$ cm^{-2}	$(1.3 \pm 0.5) \cdot 10^{13}$ cm^{-2}	frequent
V_{S2}	-	$1.5 \cdot 10^{12}$ cm^{-2}	$1.2 \cdot 10^{12}$ cm^{-2}	far less frequent than V_S
V_{2S}	far less frequent than V_S	-	-	-
V_{2S2}	-	$\sim 4 \cdot 10^{11}$ cm^{-2}	$\sim 2 \cdot 10^{11}$ cm^{-2}	-
V_{Mo}	-	$\sim 1 \cdot 10^{11}$ cm^{-2}	$9 \cdot 10^{11}$ cm^{-2}	not observed
Mo_S	-	not observed	$< 10^{11}$ cm^{-2}	-
$Mo2_{S2}$	-	not observed	not observed	-
Mo_{S2}	-	$< 10^{11}$ cm^{-2}	$< 10^{11}$ cm^{-2}	occasionally observed
V_{MoS3}	-	-	-	observed
V_{MoS2}	-	-	-	not observed
S_{Mo}	-	$< 10^{11}$ cm^{-2}	not observed	-
V_{MoS6}	-	$< 10^{11}$ cm^{-2}	not observed	occasionally observed
$S2_{Mo}$	-	$< 10^{11}$ cm^{-2}	not observed	occasionally observed

Supplementary Table 2: Summary of reported experimental observations of various types of point-like lattice defects in MoS_2, collected from refs. 12,16,18. The table only includes reports that provide information on the relative frequency of occurrence of different defect types. Where reported, the table lists areal densities of defects.

reference	probing technique	sample	V_S	comments
Hong *et al.* (ref. 16, Fig. 1d)	TEM	exfoliated natural MoS_2 monolayer	$(1.1 \pm 0.4) \cdot 10^{13}$ cm^{-2}	acceleration voltage 80 kV
		CVD-MoS_2 monolayer	$(1.3 \pm 0.5) \cdot 10^{13}$ cm^{-2}	
Zhou *et al.* (ref. 12)	TEM	CVD-MoS_2 monolayer	frequent	acceleration voltage 60 kV
Vancsó *et al.* (ref. 18)	STM	exfoliated synthetic MoS_2 monolayer	$5 \cdot 10^{12} - 5 \cdot 10^{13}$ cm^{-2}	
Lu *et al.* (ref. 19)	STM	exfoliated natural MoS_2	$3.5 \cdot 10^{10}$ cm^{-2}	
McDonnell *et al.* (ref. 20, Fig. S2c,d)	STM	exfoliated natural MoS_2	$\sim 2 \cdot 10^{11}$ cm^{-2}	in sulfur-deficient sample region. Sample exhibits variable stoichiometry, MoS_x, with $x = 1.8 - 2.3$.
Bampoulis *et al.* (ref. 21)	STM	exfoliated natural MoS_2	$(7 \pm 4) \cdot 10^{12}$ cm^{-2}	
Jeong *et al.* (ref. 22, Suppl. Info.)	STM	CVD-MoS_2 monolayer	$(4 \pm 1) \cdot 10^{11}$ cm^{-2}	MoS_2 growth protocol closely similar to that used in the present work, using the same equipment.

Supplementary Table 3: Summary of experimentally determined areal densities of sulfur monovacancies (V_S) in MoS_2, reported in the literature.[12,16,18–22]

Supplementary Note 6. Carrier type of MoS_2

In literature reports published in recent years, electrical transport measurements performed on thin MoS_2 sheets, the observed behaviour almost universally indicates n-type carriers[16,25–28]. This, however, is at variance with earlier reports from the mid-20th century, where such measurements performed on bulk MoS_2 frequently suggested p-type carriers[29–31]. Here, we briefly discuss possible reasons for this discrepancy.

Supplementary Table 4 lists several older reports on the carrier type in bulk MoS_2, and well as a selection of more recent work on bulk and thin MoS_2 samples. A suggestive trend visible in the table is that for older work on bulk MoS_2 samples, p-type transport behaviour has been reported for specimen of *natural* MoS_2 (refs. 29–31), whereas artificial MoS_2 resulted in n-type behaviour[32]. More recent work has confirmed that natural MoS_2 has variable stoichiometry MoS_x, $x = 1.8 - 2.3$, between and within samples[20,23], and it has linked n-type behavior to sulfur-deficient regions, and p-type behavior to areas of sulfur excess[20] (see also Supplementary Note 5).

Regarding thin MoS_2 samples, whereas the observation that synthetic samples produced by CVD-growth or by thermal decomposition of $(NH_4)_2MoS_4$ all exhibit n-type transport behaviour is not obvious, it is at least consistent with the earlier work on bulk artificial MoS_2 (ref. 32), and it suggests that the properties of these samples is dominated by sulfur vacancies. In contrast, it is highly anomalous that exfoliated *natural* MoS_2 samples almost universally exhibit n-type behavior, even though the source material that they are cleaved from should be expected to have a distribution of sulfur-deficient and sulfur excess regions, dominated by different types of defects respectively. While we cannot resolve this apparent contradiction conclusively, we suggest the possibility that the cleavage properties of sulfur-deficient MoS_2 might be superior to those of MoS_2 with sulfur excess (maybe due to interstitial S-atoms between layers). If thereby the fabrication of mono- or multilayer devices from natural MoS_2 with sulfur excess, expected to exhibit p-type behaviour, has only poor yield, the resulting sample preparation bias in favor of sulfur-deficient MoS_2 devices could explain the observed anomaly.

reference	sample	carrier type	comments
Regnault (ref. 29)	natural (?) MoS_2	n and p<br>p	no statement on MoS_2 source;<br>pure p-type associated with sulfur excess
Mansfield *et al.* (ref. 30)	natural MoS_2	p (22 samples)<br>n (1 sample)	
Lagrenaudie (ref. 31)	natural MoS_2	p	
Fivaz *et al.* (ref. 32)	artificial MoS_2	n (9 samples)	produced by transport reaction
McDonnell *et al.* (ref. 20)	cleaved natural MoS_2	n and p	sample exhibits variable stoichiometry, MoS_x, with $x = 1.8 - 2.3$.<br>n-type in sulfur-deficient regions,<br>p-type in excess sulfur regions.
Das *et al.* (ref. 25)	cleaved natural (?) MoS_2	n	multilayer MoS_2 on Si/SiO_2 substrate, with Sc, Ti, Ni, Pt electrodes; no statement on MoS_2 source.
Lee *et al.* (ref. 27)	cleaved natural MoS_2	n	3- and 4-layer MoS_2, encapsulated in h-BN, with graphene electrodes.
Hong *et al.* (ref. 16)	monolayer CVD-MoS_2	n	monolayer MoS_2 on Si/SiO_2 substrate, with Ti/Au electrodes, covered by HfO_2 top gate dielectric.
Ly *et al.* (ref. 28)	monolayer CVD-MoS_2	n	monolayer MoS_2 on Si/SiO_2 substrate, with Ti/Au electrodes.
Liu *et al.* (ref. 26)	synthetic MoS_2 multilayer	n	sample synthesized by thermal decomposition of $(NH_4)_2MoS_4$ on Si/SiO_2 substrate; Ti/Au electrodes.

Supplementary Table 4: Summary of electrical transport carrier types reported for MoS_2 in the literature.

Supplementary Note 7. Work function of vapour-deposited gold film

In up-to-date tabulations of materials' work functions, the work function ϕ_{Au} of gold is listed to be $5.1-5.4$ eV. Almost invariably, these values of ϕ_{Au} refer to the work of Eastman (ref. 33) and Potter and Blakely (ref. 34), who studied extremely clean gold surfaces in ultra-high vacuum. However, for gold surfaces exposed to ambient atmosphere, such as the gold substrates used to support MoS_2 samples in this work, substantially lower values for ϕ_{Au}, around $4.7-4.9$ eV, are more adequate. We briefly discuss this issue in the following.

It is important to note that the work function ϕ_{Au} represents not the properties of a bulk gold sample, but those of a specific *surface* of that sample. Thus, for crystalline gold samples, ϕ_{Au} varies between different surface reconstructions, which again differs from the value of ϕ_{Au} in the case of a polycrystalline sample[35]. Deposition of adsorbates such as oxygen and water on the gold surface also influences the work function, leading to a significant lowering of ϕ_{Au} (ref. 33).

Supplementary Table 5 lists selected values of ϕ_{Au} reported in the literature[33,34,36,37], determined by photoelectron spectroscopy and Kelvin probe surface potential measurements. It is readily apparent from the table that while clean gold surfaces free from adsorbates have high work function values, in the range $5.1-5.4$ eV, deliberate exposure to air leads to far lower values of $\phi_{\mathrm{Au}}=4.7-4.9$ eV. Since our substrates are prepared by thermal deposition of gold (50 nm), and exposed to ambient prior to MoS_2 monolayer transfer, these lower work function values are most appropriate in our case.

In order to verify the work function of our own gold substrate, we performed Kelvin probe microscopy in ambient (Hitachi E-Sweep AFM, with platinum-coated ElectriMulti75 cantilever). As reference sample we used highly oriented pyrolytic graphite (HOPG) freshly cleaved in ambient. Previous work has reported that the work function of HOPG immediately after cleaving *in air* is $\phi_{\mathrm{HOPG}}=(4.475\pm0.005)$ eV (ref. 38). Measurements of our gold substrate and the reference HOPG sample performed in immediate succession found $\phi_{\mathrm{Au}}-\phi_{\mathrm{HOPG}}=(0.35\pm0.10)$ eV, corresponding to $\phi_{\mathrm{Au}}=(4.83\pm0.11)$ eV, in agreement with earlier work[36,37].

reference	sample	ϕ_{Au}	comments
Eastman *et al.* (ref. 33)	Au	(5.1 ± 0.1) eV	photoelectron spectroscopy in ultra-high vacuum ($\sim 10^{-10}$ torr) on polycrystalline Au films ($100 - 200$ nm) deposited in-situ
Potter *et al.* (ref. 34)	Au(100)<br>Au(110)<br>Au(111)	5.47 eV<br>5.37 eV<br>5.31 eV	Kelvin probe measurements in ultra-high vacuum (reference: SnO) on single crystals, cleaned in-situ by Ar^+ bombardment at 500°C. (ϕ_{Au} evaluated in ref. 35.)
Hansen *et al.* (ref. 36)	Au, clean	$5.1 - 5.4$ eV	Kelvin probe measurements in N_2 purge gas (reference: KCl solution) on sputtered Au films (several 10's to 100's nm thickness) and Au sheets; cleaning by heating to $\sim$ 600°C in N_2 purge gas
	Au, exposed to air	$4.7 - 4.8$ eV	
Helander *et al.* (ref. 37)	Au, clean	(5.33 ± 0.05) eV	ultraviolet photoelectron spectroscopy in ultra-high vacuum ($\sim 10^{-10}$ torr) on sputtered gold films (200 nm); cleaning by Ar^+ sputtering in-situ
	Au, exposed to air	4.9 eV	

Supplementary Table 5: Summary of experimentally determined values of the work function of gold, ϕ_{Au}, reported in the literature. Refs. 33,34 provide ϕ_{Au} for very clean gold surfaces, and are quoted in most current tabulations of work function values. Refs. 36,37 study the effect of deliberate exposure of gold surfaces to air.

Supplementary References

[1] Lee, C. *et al.* Anomalous lattice vibrations of single-and few-layer MoS_2. *ACS Nano* **4**, 2695–2700 (2010).

[2] García, R. & Pérez, R. Dynamic atomic force microscopy methods. *Surf. Sci. Rep.* **47**, 197–301 (2002).

[3] Li, S.-L. *et al.* Quantitative Raman spectrum and reliable thickness identification for atomic layers on insulating substrates. *ACS Nano* **6**, 7381–7388 (2012).

[4] Chakraborty, B., Matte, H., Sood, A. & Rao, C. Layer-dependent resonant Raman scattering of a few layer MoS_2. *J. Raman Spectrosc.* **44**, 92–96 (2013).

[5] Li, H. *et al.* From bulk to monolayer MoS_2: evolution of Raman scattering. *Adv. Funct. Mater.* **22**, 1385–1390 (2012).

[6] Joo, M.-K., Kang, P., Kim, Y., Kim, G.-T. & Kim, S. A dual analyzer for real-time impedance and noise spectroscopy of nanoscale devices. *Rev. Sci. Instrum.* **82**, 034702 (2011).

[7] Hung, K. K., Ko, P. K., Hu, C. & Cheng, Y. C. A unified model for the flicker noise in metal-oxide-semiconductor field-effect transistors. *IEEE Transactions on Electron Devices* **37**, 654–665 (1990).

[8] Kirton, M. J. & Uren, M. J. Noise in solid-state microstructures: A new perspective on individual defects, interface states and low-frequency ($1/f$) noise. *Adv. Phys.* **38**, 367–468 (1989).

[9] Aitken, J., Young, D. & Pan, K. Electron trapping in electron-beam irradiated SiO_2. *J. Appl. Phys.* **49**, 3386–3391 (1978).

[10] Noh, J.-Y., Kim, H. & Kim, Y.-S. Stability and electronic structures of native defects in single-layer MoS_2. *Phys. Rev. B* **89**, 205417 (2014).

[11] Komsa, H.-P. & Krasheninnikov, A. V. Native defects in bulk and monolayer MoS_2 from first principles. *Phys. Rev. B* **91**, 125304 (2015).

[12] Zhou, W. *et al.* Intrinsic Structural Defects in Monolayer Molybdenum Disulfide. *Nano Lett.* **13**, 2615–2622 (2013).

[13] Liu, D., Guo, Y., Fang, L. & Robertson, J. Sulfur vacancies in monolayer MoS_2 and its electrical contacts. *Appl. Phys. Lett.* **103**, 183113 (2013).

[14] Guo, Y., Liu, D. & Robertson, J. Chalcogen vacancies in monolayer transition metal dichalcogenides and Fermi level pinning at contacts. *Appl. Phys. Lett.* **106**, 173106 (2015).

[15] KC, S., Longo, R. C., Addou, R., Wallace, R. M. & Cho, K. Impact of intrinsic atomic defects on the electronic structure of MoS_2 monolayers. *Nanotechnology* **25**, 375703 (2014).

[16] Hong, J. *et al.* Exploring atomic defects in molybdenum disulphide monolayers. *Nat. Commun.* **6**, 6293 (2015).

[17] Komsa, H.-P. *et al.* Two-dimensional transition metal dichalcogenides under electron irradiation: defect production and doping. *Phys. Rev. Lett.* **109**, 035503 (2012).

[18] Vancsó, P. *et al.* The intrinsic defect structure of exfoliated MoS_2 single layers revealed by Scanning Tunneling Microscopy. *Sci. Rep.* **6**, 29726 (2016).

[19] Lu, C.-P., Li, G., Mao, J., Wang, L.-M. & Andrei, E. Y. Bandgap, Mid-Gap States, and Gating Effects in MoS_2. *Nano Lett.* **14**, 4628–4633 (2014).

[20] McDonnell, S., Addou, R., Buie, C., Wallace, R. M. & Hinkle, C. L. Defect-Dominated Doping and Contact Resistance in MoS_2. *ACS Nano* **8**, 2880–2888 (2014).

[21] Bampoulis, P. *et al.* Defect Dominated Charge Transport and Fermi Level Pinning in MoS_2/Metal Contacts. *ACS Appl. Mater. Interfaces* **9**, 19278–19286 (2017).

[22] Jeong, H. Y. *et al.* Visualizing Point Defects in Transition-Metal Dichalcogenides Using Optical Microscopy. *ACS Nano* **10**, 770–777 (2016).

[23] Addou, R. *et al.* Impurities and electronic property variations of natural MoS_2 crystal surfaces. *ACS Nano* **9**, 9124–9133 (2015).

[24] KC, S., Longo, R. C., Wallace, R. M. & Cho, K. Surface oxidation energetics and kinetics on MoS_2 monolayer. *J. Appl. Phys.* **117**, 135301 (2015).

[25] Das, S., Chen, H.-Y., Penumatcha, A. V. & Appenzeller, J. High Performance Multilayer MoS_2 Transistors with Scandium Contacts. *Nano Lett.* **13**, 100–105 (2013).

[26] Liu, K.-K. *et al.* Growth of Large-Area and Highly Crystalline MoS_2 Thin Layers on Insulating Substrates. *Nano Lett.* **12**, 1538–1544 (2012).

[27] Lee, G.-H. *et al.* Highly Stable, Dual-Gated MoS_2 Transistors Encapsulated by Hexagonal Boron Nitride with Gate-Controllable Contact, Resistance, and Threshold Voltage. *ACS Nano* **9**, 7019–7026 (2015).

[28] Ly, T. H. *et al.* Misorientation-angle-dependent electrical transport across molybdenum disulfide grain boundaries. *Nat. Commun.* **7**, 10426 (2016).

[29] Regnault, F., Aigrain, P., Dugas, C. & Jancovici, B. Sur les propriétés semi-conductrices de la molybdénite. *C. R. Acad. Sci .* **235**, 31–32 (1952).

[30] Mansfield, R. & Salam, S. Electrical properties of molybdenite. *Prc. Phys. Soc. B* **66**, 377 (1953).

[31] Lagrenaudie, J. Comparaison des composés de la famille de MoS_2 (structure et propriétés optiques et électriques). *J. Phys. Radium* **15**, 299–300 (1954).

[32] Fivaz, R. & Mooser, E. Mobility of charge carriers in semiconducting layer structures. *Phys. Rev.* **163**, 743 (1967).

[33] Eastman, D. Photoelectric work functions of transition, rare-earth, and noble metals. *Phys. Rev. B* **2**, 1 (1970).

[34] Potter, H. & Blakely, J. LEED, Auger spectroscopy, and contact potential studies of copper- gold alloy single crystal surfaces. *J. Vac. Sci. Technol.* **12**, 635–642 (1975).

[35] Michaelson, H. B. The work function of the elements and its periodicity. *J. Appl. Phys.* **48**, 4729–4733 (1977).

[36] Hansen, W. N. & Johnson, K. B. Work function measurements in gas ambient. *Surf. Sci.* **316**, 373–382 (1994).

[37] Helander, M., Greiner, M., Wang, Z. & Lu, Z. Pitfalls in measuring work function using photoelectron spectroscopy. *Appl. Surf. Sci.* **256**, 2602–2605 (2010).

[38] Hansen, W. N. & Hansen, G. J. Standard reference surfaces for work function measurements in air. *Surf. Sci.* **481**, 172–184 (2001).
